# Postpartum depressive symptoms and its association to daytime sleepiness and restless legs during pregnancy

**DOI:** 10.1186/s12884-016-0917-9

**Published:** 2016-06-06

**Authors:** Maria Sarberg, Marie Bladh, Eva Svanborg, Ann Josefsson

**Affiliations:** Department of Obstetrics and Gynaecology, University Hospital and Linköping University, SE−581 85, Linköping, Sweden; Department of Clinical and Experimental Medicine, Linköping University, Linköping, Sweden; Department of Clinical Neurophysiology, Linköping University, Linköping, Sweden

**Keywords:** Postnatal depression, Sleep, Restless legs syndrome, Pregnancy, Epworth Sleepiness Scale

## Abstract

**Background:**

Postpartum depression is a common condition, which consequences might be harmful for both mother and child. Since sleep and depression are closely related it is possible that women who suffer from sleep related problems during pregnancy are more likely to develop depression in the postpartum period. This study aims to investigate the possible association between depressive symptoms in the postpartum period and sleep related problems during pregnancy.

**Methods:**

In this study 293 women in the last trimester of pregnancy answered a questionnaire about symptoms of restless legs, snoring and daytime sleepiness. They also completed the Epworth Sleepiness Scale (ESS). The same women were screened for depressive symptoms using the Edinburgh Postnatal Depression Scale (EPDS) four to ten weeks after giving birth. Additional information about social data, pregnancy and delivery was received from the medical charts.

**Results:**

Women with postpartum depressive symptoms had higher prevalence of excessive daytime sleepiness defined as ESS score ≥10 (OR 3.84, CI 1.57–9.39), and restless legs syndrome (OR 2.837 CI 1.18–6.84) in last trimester of pregnancy, when adjusted for socio-demographic factors and obstetric risk factors. No association was found between postpartum depressive symptoms and snoring.

**Conclusions:**

Depressive symptoms after childbirth are preceded by sleep related problems such as daytime sleepiness and restless legs, already during pregnancy. The results from Epworth Sleepiness Scale and a questionnaire concerning Restless Legs Syndrome completed during pregnancy might be a valuable contribution for detecting women at risk for postpartum depression, enabling preventive interventions.

## Background

Postpartum depression (PPD) is a major depression that begins in or extends to the period after giving birth. It affects 10–15 % of all new mothers [1, 2]. The consequences of depression in the postnatal period can be harmful for both mother and child since it can affect the mother-child relationship, the mothers’ self-esteem and her social and personal adjustment to her new role. Studies also show an impact on the cognitive and emotional development of the child [3, 4].

Sleep and depression are closely related. It is known that up to 90 % of all persons suffering from major depression have symptoms of subjective sleep disturbances [5]. Additionally, there is evidence that sleep alterations precede the onset of depression [6–8]. It is well known that sleep changes during pregnancy with impaired quality of sleep, frequent nocturnal awakenings, fewer hours of sleep and lower sleep efficacy [9, 10]. We also found that sleep disturbing conditions such as snoring and restless legs syndrome increase during pregnancy [11, 12]. Restless legs syndrome (RLS) is characterized as paresthesia or dysesthesia, usually in the legs, causing a desire to move the limbs with immediate temporary relief by activity. The symptoms are aggravated at rest and in the evening or early night [13], which might cause insomnia and insufficient sleep length. There is a known correlation between RLS and major depression in the general population [14, 15]. A newly published paper indicates that women with RLS onset before pregnancy had an increased risk of both antenatal depression and PPD [16], but there is no other evidence that RLS during pregnancy affects the prevalence of PPD.

There are a number of studies that show an association between sleep during pregnancy and depression or depressive symptoms before delivery [17–19] and there is also a large survey describing an association between poor sleep quality postpartum and depression [20]. A few studies have also explored the relationship between PPD and sleep during pregnancy, but they report diverging results and use different methods for measuring sleep associated factors [21–25]. There is evidence that subjective perception of sleep, more than objective sleep quality is associated with postpartum depressive symptoms [26, 27]; however this has not been evaluated using validated screening tools.

The aim of this study was to compare the frequencies of sleep related problems during pregnancy among women with the prevalence of depressive symptoms in the postpartum period. The sleep related problems considered were restless legs, daytime sleepiness, morning fatigue and snoring as measured by validated and convenient screening instruments.

## Methods

### Study population

Pregnant women who took part in a study on sleeping disorders and restless legs during pregnancy [11] were recruited at a Swedish antenatal care clinic (ACC) in the year 2007. The Swedish antenatal care program reaches almost 100 % of all pregnant women and is free of charge [28]. Women with diabetes mellitus, neurological disease, drug abuse, hypertension or poor knowledge of the Swedish language were excluded. After receiving written and oral information 351 women participated in this study. Written informed consent was obtained from each participant. In the 3rd trimester of pregnancy the contributing women were given the Epworth Sleepiness Scale (ESS) and an additional questionnaire about their sleep. At the routine postpartum check-up visit around ten weeks after delivery, the women were also screened for PPD as part of the normal routine.

### Questionnaires

The questionnaires distributed in the 3rd trimester of pregnancy consisted of the ESS, and also questions about morning fatigue, daytime sleepiness, snoring and symptoms of restless legs.

### Screening for daytime sleepiness

Epworth Sleepiness Scale (ESS) [29] was used to screen for sleepiness. It is a standardized and validated instrument for measuring sleepiness among patients with snoring and obstructive sleep apnoea, but is also validated for evaluation of sleepiness due to other causes [30] and for use during pregnancy [31]. In the ESS the probability of falling asleep for eight different situations in daily life is ranked on a scale of increasing probability from 0 to 3. Maximum score is 24; a score ≥10 is usually considered abnormal, indicating excessive daytime sleepiness (EDS). The women were also asked to rate their experience of morning fatigue and daytime sleepiness answering the questions “Do you feel tired and badly rested when you wake up at morning?” and “Do you feel sleepy during the day?”, using the alternatives “always”, “often”, “sometimes”, “seldom” or “never”. The women who answered “often” or “always” on these questions were identified as suffering from “morning fatigue” and “daytime sleepiness” respectively.

### Screening for snoring

The women rated their snoring using the alternatives “always”, “often”, “sometimes”, “seldom” or “never” on a daily basis. Women who stated that they snored “often” or “always” were considered habitual snorers.

### Screening for RLS

To identify women with RLS the four separate questions set by the International RLS Study Group for diagnosing restless legs syndrome in epidemiological studies [32] was used: occurrence of unpleasant sensations in the legs combined with need for movement, if these sensations are chiefly present at rest and decreased by movement, if the sensations is worse in the evenings or during the nights compared to the mornings and how often the sensations occur. Women who answered positively to the first three questions and experienced the symptoms at least once per month were considered sufferers from restless legs.

### Screening for depressive symptoms

At the postpartum check-up the women were assessed with the Edinburgh Postnatal Depression Scale (EPDS). The EPDS is a widely used instrument specifically designed for detecting PPD [33]. The original version in English is validated [34], as well as the Swedish translation used in this study [35]. It is a ten item self-report scale measuring common symptoms of depression. Each item is scored on a four point scale (0–3) and rates the intensity of depressive symptoms during the previous seven days. The maximum score is 30. In order to find all actual major depressions, Cox et al. proposed a cut-off level ≥10 to reduce detection failure in the postpartum period [33]. By selecting this threshold, the sensitivity for detection of major depression increased to almost 100 % and the specificity to 82 % [36]. In this study the cut-off level of ≥10 was used.

### Medical and social data

Data concerning the characteristics of the women and their pregnancies which were considered possible risk factors for developing PPD were taken from the Swedish standardized antenatal and delivery records. Variables taken in consideration were: age, marital status, occupation, parity, body mass index at first visit to ACC (mean gestational week 11.2, SD 1.6), use of alcohol, illegal drugs or tobacco, prior PPD, current depressive disorder, medication, intercurrent somatic disease, stressful life events during current pregnancy, number of appointments to obstetric medical staff during pregnancy, fear of delivery, mode of delivery and diagnosed complications of pregnancy and/or delivery. For classification of occupations the ISCO −88 system was used [37], and the occupations was divided into two skill levels according to the ISCO −88. Women who did not fit into one of these groups (students, unemployed or on permanent sick leave) were referred to as a third group.

### Statistical analysis

Continues variables were analysed using student *T*-test and dichotomized variables using Pearson Chi square test. Data presented in Tables 2–4 were analysed using single and multiple logistic regression with EPDS groups as dependent variable and each factor presented in the tables were entered as independent variables, each tables modelled separately. Data in Tables 2–4 were also adjusted for socio-demographic background data from Table 2. Data in Table 4 were adjusted for all variables in Table 2 and the variables identified as risk factors for high prevalence of postpartum depressive symptoms in Table 3. The significance level was set to 5 % (two-sided) in all tests. The statistical software IBM SPSS 21.0 (IBM SPSS Inc., Armonk, NY) was used.

## Results

In total, 351 women answered the questionnaire about sleep in the 3rd trimester (mean gestational week 34.4, SD 0.6). Fifty-eight of the women (16.5 %) were not screened for PPD at postnatal check-up. The reasons for the drop-outs are shown in Table 1. These 58 women did not differ from the remaining women in terms of age, BMI, parity or prevalence of RLS and snoring. However, the women who were screened for PPD had higher scores on ESS during pregnancy (9.0 vs. 7.7, *p*-value 0.017).

**Table 1:** Causes for absence of PPD screening

Non-attendance to postpartum check-up	18
Attendance, but not screened for unknown reasons	38
Other^a^	2
Total	58

^a^Death of neonate, or PPD diagnosed before postpartum check-up

The 293 women who underwent screening for PPD (mean 9.2 weeks after giving birth, SD 5.8) were included in the statistical analysis. They had a mean EPDS score of 4.96 (range 0–22, SD 3.42). Twenty-nine women (9.9 %) had an EPDS score ≥10.

### Medical and social data

Demographic and medical background data are shown in Table 2. No women claimed that they used alcohol or illegal drugs at inclusion in the study. Twenty-seven women had a history of any psychiatric disease before pregnancy, but none of them had suffered from psychotic disease. These women did not have significantly higher EPDS-score (Table 2). Women with previous PPD had an increased risk for high prevalence of postpartum depressive symptoms after their current pregnancy, however the confidence interval was very wide and the association was no longer significant when it was adjusted for background factors (Table 2).

**Table 2:** Background on studied women including unadjusted and adjusted odds ratio for EPDS ≥10

		EPDS < 10		EPDS ≥ 10				
		*n* = 264		*n* = 29				
		*n*	%		%	*p*-value*	OR (95 % CI)	Adj. OR^a^ (95 % CI)
Age (years. mean/SD)		30.1/4.28		29.9/4.95		0.822	0.99 (0.90–1.08)	1.03 (0.94–1.13)
Relationship status								
	Partnered	261	98.9	28	96.6	0.308	Reference	Reference
	Single	3	1.1	1	3.4		3.11 (0.31–30.88)	1.62 (0.14–20.52)
Parity	Primi	127	48.1	14	48.3	0.986	1.01 (0.47–2.17)	1.26 (0.52–3.09)
	Multi	137	51.9	15	51.7		Reference	Reference
BMI at start of pregnancy (kg/m^2^; mean/SD)		24.0/3.92		24.0/3.60		0.792	1.01 (0.91–1.11)	1.09 (0.46–2.55)
	Normal	182	69.7	18	62.1	0.397	Reference	Reference
	Overweight	79	30.3	11	37.9		1.41 (0.64–3.12)	1.17 (0.51–2.70)
Smoking								
	Yes	11	4.2	1	3.4	0.853	1.22 (0.15–9.78)	0.63 (0.07–5.48)
	No	253	95.8	28	96.6		Reference	Reference
Education/occupation								
	High skill	162	61.4	10	34.5	0.019	Reference	Reference
	Low skill	63	23.9	11	37.5		2.83 (1.15–6.99)	3.11 (1.17–8.29)
	Other/non	39	14.8	8	27.6		3.32 (1.23–8.97)	3.08 (1.05–9.04)
Any psychiatric disease prior to pregnancy^b^								
	Yes	22	8.3	5	17.2	0.115	2.29 (0.74–6.60)	1.16 (0.29–4.63)
	No	242	91.7	24	82.8		Reference	Reference
Previous PPD^c^								
	Yes	2	1.5	2	13.3	0.001	10.39 (1.35–79.94)	5.91 (0.44–79.45)
	No	135	98.5	13	86.7		Reference	Reference

*p-value based on student *T*-test or Chi square test

^a^Adjusted for all the variables in Table 2

^b^Any psychiatric disorder prior to pregnancy: Depression, anxiety disorder, eating disorder or prior PPD

^c^Analysis based on multiparous women

Medical and social events during pregnancy and labour are presented in Table 3. These data were adjusted for all background data displayed in Table 2. Eight women experienced depression during their pregnancy, two of them had received this diagnosis already before becoming pregnant. One woman started taking anti-depressive medication during her pregnancy and four underwent non-directive counselling. Two women with EPDS score ≥10 had experienced stressful life events during their pregnancies, which were related to present or past relationships. All 18 women who stated that they were afraid of delivery underwent a special non-directive counselling for this. Of the 51 women who did not have a normal delivery, 17 were delivered with vacuum extraction, 20 with elective caesarean section and 14 with acute caesarean section. Only 19 women did not breast feed at all at the postpartum check-up, and the difference between the women with high vs. normal EPDS score was not statistical significant (OR = 1.095, CI 0.240–5.007) (data not shown). Since most of the analysed obstetric complications are rare, and the group with EPDS ≥ 10 is relatively small, the confidence intervals for many of the obstetric outcomes are large, indicating a statistical uncertainty.

**Table 3:** Pregnancy and delivery related data on studied women including unadjusted and adjusted odds ratio for EPDS ≥10

		EPDS < 10		EPDS ≥ 10				
		*n* = 264		*n* = 29				
		*n*	%	*n*	%	*p*-value*	OR (95 % CI)	Adj. OR^a^ (95 % CI)
Depression during current pregnancy	Yes	5	1.9	3	10.3	0.008	5.98 (1.35–26.42)	8.81 (1.07–72.39)
	No	259	98.1	26	89.7		Reference	Reference
Current antidepressive medication during pregnancy^b^								
	Yes	3	1.1	1	3.4	0.308	3.11 (0.31–30.88)	1.71 (0.09–32.38)
	No	261	98.9	28	96.6		Reference	Reference
Intercurrent somatic disease^c^								
	Yes	13	4.9	2	6.9	0.647	1.43 (0.31–30.88)	1.29 (0.25–6.28)
	No	251	95.1	27	93.1		Reference	Reference
Stressful life event during pregnancy								
	Yes	8	3.0	2	6.9	0.276	2.37 (0.48–11.73)	2.03 (0.38–11.54)
	No	256	97.0	27	93.1		Reference	Reference
Number of visits during pregnancy^d^(no of visits mean/SD)		10.2/2.4		11.2/2.9		0.032	1.16 (1.01–1.32)	1.16 (0.99–1.36)
Fear of delivery								
	Yes	16	6.1	2	6.9	0.859	1.15 (0.25–5.26)	0.89 (0.16–4.88)
	No	248	93.9	27	93.1		Reference	Reference
Complication during pregnancy^e^								
	Yes	58	22,0	6	20,7	0.874	0.93 (0.36–2.38)	0.74 (0.26–2.05)
	No	206	78,0	23	79,3		Reference	Reference
Premature birth (<36 + 6)^f^								
	Yes	3	1.1	3	10.3	0.001	10.04 (1.93–52.29)	13.07 (2.08–82.26)
	No	261	98.9	26	89.7		Reference	Reference
Normal delivery								
	Yes	221	83.7	21	72.4	0.128	Reference	Reference
	No	43	16.3	8	27.6		1.96 (0.81–4.71)	1.84 (0.71–4.76)
Complication during or after delivery^g^								
	Yes	33	12.5	4	13.8	0.842	1.12 (0.37–3.42)	1.25 (0.39–3.94)
	No	231	87.5	25	86.2		Reference	Reference

*p-value based on student *T*-test or Chi square test

^a^Adjusted for all the variables in Table 2

^b^SSRI or SNRI

^c^Trombophilic disorder, inflammatory bowel disease, hypo- or hyperfunction of the thyroid gland, epilepsy, prolactinoma and cerebral shunt

^d^Total number of visits during pregnancy to a midwife or physician, except for the visit when they were giving birth

^e^Severe hyperemesis, contractions (need for sick leave >2 weeks), back or pelvic girdle pain (need for medical counselling), gestational diabetes, abnormal vaginal bleeding (hospitalization), carpal tunnel syndrome, cholestasis of pregnancy or thromboembolic disease

^f^Between gestational week 34–36

^g^Bleeding >1000 ml, urine retention, rupture including anal sphincter, pneumonia or postpartum endometritis

### Sleepiness related data

The mean ESS score in the entire group of women was 8.98 (SD 3.82). Women with normal EPDS score had mean ESS 8.83 (SD 3.79) and women with EPDS ≥10 had mean ESS 10.34 (SD 3.86). The difference between the groups was statically significant (*p* = 0.043). When defining daytime sleepiness as ESS ≥ 10, the sensitivity of this test for predicting EPDS ≥ 10 was 59 % and the positive predictive value 16 %. The specificity was 66 % and the negative predictive value was 94 %.

Sleep related data are presented in Table 4. These data are adjusted for the background data in Table 2 and also for the statistically significant variables in Table 3. The difference in daytime sleepiness was significant between the women with high vs. normal EPDS score, both defined as ESS score ≥10 and when asked as a yes or no question (“Do you feel tired and badly rested when you wake up in the morning?”). Also morning fatigue and restless legs in 3rd trimester of pregnancy showed significant association with high postpartum EPDS score, but not snoring during pregnancy (OR shown in Table 4).

**Table 4:** Sleep related data on studied women including unadjusted and adjusted odds ratio for EPDS ≥10

		EPDS < 10		EPDS ≥ 10					
		*n* = 264		*n* = 29					
		*n*	%		%	*p*-value*	OR (95 % CI)	Adj. OR^a^ (95 % CI)	Adj. OR^b^ (95 % CI)
Excessive daytime sleepiness (ESS ≥ 10)									
	Yes	91	34.5	17	58.6	0.010	2.69 (1.23–5.88)	2.77 (1.23–6.23)	3.84 (1.57–9.39)
	No	173	65.5	12	41.4		Reference	Reference	
Daytime sleepiness									
	Yes	75	30.1	15	57.7	0.004	3.16 (1.39–7.21)	3.48 (1.42–8.49)	3.29 (1.30–8.33)
	No	174	69.9	11	42.3		Reference	Reference	
Morning fatigue^c^									
	Yes	58	22.1	14	50.0	0.001	3.52 (1.59–7.80)	3.55 (1.50–8.42)	3.17 (1.23–7.87)
	No	204	77.9	14	50.0		Reference	Reference	
Habitual snoring									
	Yes	52	20,0	6	23.1	0.710	1.20 (0.46–3.14)	1.03 (0.95–1.16)	0.98 (0.31–3.11)
	No	208	80,0	20	76.9		Reference	Reference	
Restless legs symptoms									
	Yes	75	28.4	14	48.3	0.027	2.35 (1.08–5.11)	2.50 (1.09–5.71)	2.84 (1.18–6.84)
	No	189	71.6	15	51.7		Reference	Reference	

*p-value based on student *T*-test or Chi square test

^a^Adjusted for all the variables in Table 2

^b^Adjusted for all variables in Table 2 and statistically significant variables from the multivariate analysis in Table 3

^c^3 women did not answer this question

## Discussion

This study shows an association between high prevalence of depressive symptoms in the postpartum period and high ESS score, daytime sleepiness, morning fatigue and restless legs symptoms in last trimester of pregnancy. There is evidence that disturbed sleep precedes depressive symptoms in the general population [5] and that poor sleep quality in early pregnancy might contribute to antepartum depressive symptoms during pregnancy [17]. Therefore it is likely that poor sleep already during pregnancy increases vulnerability for development of PPD. This is of importance as PPD is a common condition causing severe suffering for the new mother and her family, and all possibilities to prevent this is valuable. Almost all Swedish pregnant women have regular contacts with the ACC which enables regular screening for disturbed sleep, and information and counselling about good sleep as prevention for later depression. There is evidence that sleep educational programs might decrease postpartum depressive symptoms [38]. A newly published Cochrane report regarding PPD [39] appoints that interventions targeting women at risk are the most beneficial to prevent postpartum depressive symptoms.

In Sweden almost 100 % of all pregnant women attend the antenatal care program. The present survey constitute a large number of consecutively recruited women who were followed during and after pregnancy to avoid selection bias. However, there was a drop-out of 58 women (16.5 %) who never answered the EPDS which is a possible weakness in the study.

Validated instruments for measuring depressive symptoms (EPDS), sleepiness (ESS) and RLS were used. We intentionally chose to evaluate possible sleep disturbances by use of the ESS, despite the fact that this does not actually say anything about objective sleep. This is in line with results from previous studies, showing that the association between postpartum mood and the subjective perception of sleep is stronger than between postpartum mood and objective sleep quality and duration [26, 27]. The use of a screening tool, only taking a few minutes to complete, might also contribute to a larger study population by making participation in the study more attractive for both women and their midwives. It might also make implementation of the results easier, since the same screening tool can be used in the clinic as part of the normal antepartum program. But, since poor sleep and tiredness might be symptoms of an already existing depression, it is possible that a high ESS score during pregnancy could actually indicate an antepartum depression.

The prevalence of EPDS ≥10 was 9.9 % among the women in our study, which is somewhat lower than in other research [1]. On the other hand, the identified risk factors in this study for a high prevalence of depressive symptoms after delivery (low education level, previous PPD, depression during current pregnancy and high number of visits to medical staff during pregnancy) corresponds well with previous knowledge [40]. We also found that women with EPDS ≥10 experienced more daytime sleepiness and morning fatigue than the other women. There are some smaller studies reporting similar associations between poor sleep quality during pregnancy and development or recurrence of PPD or postpartum depressive symptoms [22, 23, 26]. Furthermore, there is a larger Portuguese study reporting association between insomnia in the last trimester of pregnancy and postpartum depressive symptoms, but not with PPD [24]. However, none of these studies used a validated screening tool such as ESS for daytime sleepiness.

ESS might be a valuable screening tool to objectively verify daytime sleepiness due to sleeping problems during pregnancy. Our data suggests that high ESS scores during pregnancy also predict development of PPD. In our study, the sensitivity of ESS (cut off level ≥10) for predicting PPD was 59 % and the sensitivity was 66 %. This indicates that screening for daytime sleepiness during pregnancy can be a contribution, but not a perfect screening instrument for predicting PPD.

We also found that prevalence of RLS in last trimester of pregnancy is associated with depressive symptoms in the postpartum period, which confirms the association between RLS onset before pregnancy and both antenatal depression and PPD described by Wesström et al. [16]. We found no association between snoring during pregnancy and postpartum depressive symptoms, although O'Brien et al. have reported maternal snoring as a risk factor for prenatal depressive symptoms [41].

The major strength of this survey is its size and reliability. Our results do not clarify whether sleep disturbance or depressive symptoms occur first. If the women had completed the ESS and the EPDS both at the 3rd trimester visit and at the postpartum check-up this could have added extra strength to the study.

## Conclusions

The aetiology of postpartum depression is most likely multifactorial. However, daytime sleepiness already during pregnancy increases the risk of postpartum depressive symptoms. This is true particularly when considering excessive daytime sleepiness during pregnancy, identified as ESS score ≥10. This association suggests that ESS scoring during pregnancy might be a valuable contribution in screening for a high risk of developing PPD. Also the presence of RLS during late pregnancy is associated with postpartum depressive symptoms.

## Abbreviations

ACC, antenatal care clinic; ESS, Epworth Sleepiness Scale; EPDS, Edinburgh Postnatal Depression Scale; PPD, postpartum depression; RLS, restless legs syndrome
